# Frequency of Extreme Heat Event as a Surrogate Exposure Metric for Examining the Human Health Effects of Climate Change

**DOI:** 10.1371/journal.pone.0144202

**Published:** 2015-12-07

**Authors:** Crystal Romeo Upperman, Jennifer Parker, Chengsheng Jiang, Xin He, Raghuram Murtugudde, Amir Sapkota

**Affiliations:** 1 Maryland Institute for Applied Environmental Health, University of Maryland School of Public Health, College Park, Maryland, United States of America; 2 Marine Estuarine Environmental Science Program, University of Maryland, College Park, Maryland, United States of America; 3 National Center for Health Statistics, Centers for Disease Control and Prevention, Hyattsville, Maryland, United States of America; 4 Department of Epidemiology and Biostatistics, University of Maryland School of Public Health, College Park, Maryland, United States of America; 5 Department of Atmospheric and Oceanic Science, University of Maryland, College Park, Maryland, United States of America; University of Washington, UNITED STATES

## Abstract

Epidemiological investigation of the impact of climate change on human health, particularly chronic diseases, is hindered by the lack of exposure metrics that can be used as a marker of climate change that are compatible with health data. Here, we present a surrogate exposure metric created using a 30-year baseline (1960–1989) that allows users to quantify long-term changes in exposure to frequency of extreme heat events with near unabridged spatial coverage in a scale that is compatible with national/state health outcome data. We evaluate the exposure metric by decade, seasonality, area of the country, and its ability to capture long-term changes in weather (climate), including natural climate modes. Our findings show that this generic exposure metric is potentially useful to monitor trends in the frequency of extreme heat events across varying regions because it captures long-term changes; is sensitive to the natural climate modes (ENSO events); responds well to spatial variability, and; is amenable to spatial/temporal aggregation, making it useful for epidemiological studies.

## Introduction

Climate change is expected to cause approximately 250,000 deaths per year between 2030 and 2050 with direct damage costs totaling $ 2–4 billion USD per year by 2030 [1]. Chronic diseases, that may be exacerbated by climate change, disproportionately affect more vulnerable populations—including children, older adults, the socially isolated, and those with mental health issues [2]. Epidemiological investigation of the impact of climate change on human health is hindered by the differing temporal scale of the primary exposure of interest (climate change: decadal scale) and health outcomes that have varying sensitive time windows (days to years) in epidemiological studies that are based on a few years of data [3,4]. There is a need for a set of suitable exposure metrics that can capture the subtle attributes of changing climate (e.g. frequency, duration, and intensity of extreme events that are expected to rise), and would allow for comparisons across different geographical locations and time periods. Such an exposure metric should have enough flexibility for temporal aggregation to meet the needs of different types of epidemiological studies. Furthermore, for national health studies, there is a need for spatial compatibility, as meteorological data are often available at station level or a specific grid, whereas, national health data (e.g., behavioral risk factor surveillance system (BRFSS), Centers for Medicare and Medicaid Services (CMS), healthcare cost and utilization project (HCUP), CDC’s Public Health Tracking data) are commonly available at zip code, county or state levels, often with a non-uniform spatial resolution.

Public health researchers are increasingly using temperature measures (maximum, minimum, heat index, and apparent temperature), and heat wave episodes to identify the acute health outcomes associated with weather. While the linkage of frequency and intensity of heat waves with acute health outcomes provide important information about long-term climate trends and health, heat waves are still relatively uncommon in most locations in the US [5,6], limiting the generalizability of study results across locations and over time. Moreover, heat wave measurements are designed to capture physical phenomenon: most common definitions include a certain number of consecutive days exceeding a location-specific threshold [7]. Hence, by definition, heat wave does not capture isolated days where temperatures may have been high and, possibly, affecting health. Extreme heat events, on the contrary, will capture such isolated event.

For chronic health outcomes, the meaningful window of exposure may vary from months to several years. Not surprisingly, the relationships between climate change and chronic health outcomes are less understood than acute health outcomes, such as mortality or an emergency department (ED) visit, owing to the difficulty in defining appropriate exposure metrics that characterize underlying and long-term climate change at varying temporal and spatial resolutions and that are appropriate for chronic health outcomes [8,9].

In this paper we describe an indicator designed to capture exposure to climate variability and change at a spatial scale that is consistent with publically available county-level health outcome data. This indicator, henceforth referred to as an exposure metric or “*extreme heat event*”, captures positive anomaly derived using distributions of county- and month-specific climatology using a 30-year reference period (1960–1989). Although both extreme heat and extreme cold are important, this manuscript focuses exclusively on extreme heat because warmer temperature is associated with etiology of many infectious (higher rates of pathogen replication) as well as chronic diseases (increases in concentration of pollutant such as ozone). The exposure metric enables users to look at spatial and temporal changes over time using location specific baselines and serves as an additional resource to investigate the potential relationships between climate change and human health. We tabulate this exposure metric by time period, season, Census division, and 2006 urban-rural classification, documenting how the exposure metric is amenable to spatial and temporal aggregation across factors that are known to be associated with variability in temperature. Finally, we evaluate this exposure metric by assessing its correspondence to the different phases of *El*-*Ni*ñ*o-Southern Oscillation* (ENSO), a natural oscillation patterns that affect the weather phenomenon in the continental US and other parts of the world.

## Materials and Methods

Meteorological data were acquired from the National Climatic Data Center (NCDC) branch of the National Oceanic and Atmospheric Association (NOAA) that maintains the world’s largest archive of meteorological data from the past 150 years. The data used to develop the metric are archived in two broad categories: DSI-3200 and DSI-3210. The DSI-3210 network is a smaller subset of DSI-3200 stations that collect several additional weather variables besides temperature and precipitation (e.g., barometric pressure, wind speed, wind direction), but has a poor spatial coverage. Therefore we chose the DSI-3200 database that contains approximately 8,000 active stations, with up to 23,000 stations for various years. The stations cover all 50 states plus Puerto Rico, US Virgin Islands and Pacific Island territories. Each dataset underwent quality control measures through both automated and manual edits by the NCDC, which consisted of internal consistency checks and evaluation against adjacent stations [10]. To develop and evaluate the metric, we used climate data for the 48 contiguous states and the District of Columbia. The county boundaries used for all years were defined by the 2000 Federal Processing Standards (FIPS) codes.

For *Urban-Rural* status, we used the 2006 county level National Center for Health Statistics (NCHS) Urban-Rural Classification Scheme. The 2006 NCHS urban-rural classification scheme was developed for use in studying and monitoring health disparities across the urban-rural continuum. The 2006 scheme consists of four levels of metropolitan counties (large central, large fringe, medium and small metro) and two levels of nonmetropolitan counties (micropolitan and non-core). This scheme is based on the December 2005 Office of Management and Budget delineations of county classification, Metropolitan Statistical Area (MSA) and principal city, MSA population-size cut points, and classification rules formulated by NCHS [11]. In congruence with other studies [12–17], we opted to use this relatively recent classification scheme for the complete 51 years of data (1960–2010). However, because the 2006 scheme is not as accurate for earlier time periods as it is for more recent time periods [11], we also tabulated the extreme heat events using a 1990 urban rural classification [18] to conduct a sensitivity analysis.

The census division classification used in this study consists of groups of contiguous states as defined by the US Bureau of the Census (as: New England–CT, ME, MA, NH, RI, VT; Middle Atlantic–NJ, NY, PA; South Atlantic–DE, DC, FL, GA, MD, NC, SC, VA, WV; East South Central–AL, KY, MS, TN; West South Central–AR, LA, OK, TX; East North Central- Il, IN, MI, OH, WI; West North Central–IA, KS, MN, MO, NE, ND, SD; Mountain–AZ, CO, ID, MT, NV, NM, UT, WY; and Pacific–CA, WA, OR). Seasons were defined as: Winter–December, January, and February; Spring–March, April, and May; Summer–June, July, and August; and Autumn–September, October, and November.

*El*-*Ni* **ñ** *o-Southern Oscillation* (ENSO) indicator data—Oceanic Ni**ñ**o Index (ONI)—were obtained from the National Oceanic and Atmospheric Administration (NOAA), National Weather Service Climate Prediction Center. The Climate Prediction Center is a coordinated program that monitors, assesses and predicts climate phenomena and their linkage to weather events. Warm and cold episodes are based on a threshold of +/- 0.5°C for the Oceanic Niño Index (ONI)—a 3-month running median anomalies in the sea surface temperature in the Niño 3.4 region (5°N-5°S, 120°-170°W). The threshold values are based on centered 30-year base periods and are updated every 5 years. El Niño and La Niña episodes are defined when the threshold is met for a minimum of 5 consecutive over-lapping seasons. This ENSO indices data were used to categorize the months as La Niña, El Niño and Neutral months [19].

### Extreme Heat Events

We assigned daily maximum temperature for all counties using the following rules: 1) average of daily maximum temperatures from all stations within the county, 2) if no station data were available from the county, the daily maximum temperature used was from the closest available station within a 20 km radius of the county boundary, and 3) a missing value was assigned if the previous two criteria were not met. In the complete dataset of observations, 99% of all counties had less than 1.5% missing data and there was no spatial pattern to the location of missing data. To compute extreme heat events, we used 1960–1989 as a *reference period*. This time period was chosen because the weather data were recorded consistently with current methods of NCDC measurement and the 30-year time period is generally accepted as the epoch (per the IPCC report) to represent the standardization of a climate regime [20]. For each county within the continental US, we compiled daily maximum temperatures (*T*_*max*_) by calendar months (e.g., Jan 1^st^ to Jan 31^st^). For the 30-year reference period with no missing data, the total number of values would be approximately 900 observations (30 years by ~30 days in a month) for each county and calendar month. Using this distribution of daily *T*_*max*_, we calculated the month specific 95^th^ percentile thresholds for each county. Using this cutoff value, we computed the calendar month and year specific extreme heat events for each county as:

$$E_{jk}=\sum_{i}\;I_{ijk}\;where\;I_{ijk}=\{\begin{array}{c} \;1,\;if\;T_{ijk-max}\;>\;T_{jk-95}\\ \;0,\;if\;T_{ijk-max}\;\leq \;T_{jk-95} \end{array}$$ (1)

where *E*_*jk*_ is the total number of extreme heat events for county *j* in calendar month *k*; *T*_*ijk-max*_ is the daily maximum temperature (*T*_*max*_) in county *j* for day *i* of calendar month *k*; *T*_*jk-95*_ is the 95^th^ percentile *T*_*max*_ value for county *j* for calendar month *k* for the 1960–1989 period; and *I*_*ijk*_ represents the indicator of whether or not *T*_*ijk-max*_ is greater than *T*_*jk*−95_.

### Evaluation

The units of analysis for our evaluation of the indicator were the annual and monthly total number of events; these are the metrics that are referenced throughout the paper. All covariates of interest were defined at the county, year and month levels. We computed descriptive statistics of the spatial (2006 urban-rural classification, Census division) and temporal (seasonal, decadal) characteristics. Additional descriptive statistics were calculated for ENSO periods. After checking the normality assumption, comparisons of means were performed using one-way analysis of variance (ANOVA) and post-hoc Tukey’s honest significant difference (HSD) tests [21]. We further investigated the temporal and spatial dependency of the exposure metric using negative binomial generalized estimation equation (GEE) models [22,23]. The year and monthly total extreme heat event anomalies in each county were modeled as a function of seasonality, ENSO, 2006 urban-rural classification, and Census division. We identified findings as statistically significant with a p-value <0.05. Most statistical analyses were performed using SAS 9.3 (SAS Institute, Cary, NC). In particular, PROC GENMOD was used to fit the negative binomial GEE models using a first-order autoregressive covariance structure. The exponent of the estimated regression coefficients was calculated to estimate the percent change in the mean response (number of extreme heat events) associated with changes in the covariates. Regression maps were created using ArcGIS 10 (esri, Redlands, CA) to display the county level regression parameter estimate for the impact of ENSO on the number of extreme heat events after adjusting for seasonal and 2006 urban-rural classification.

## Results

**Fig 1:**
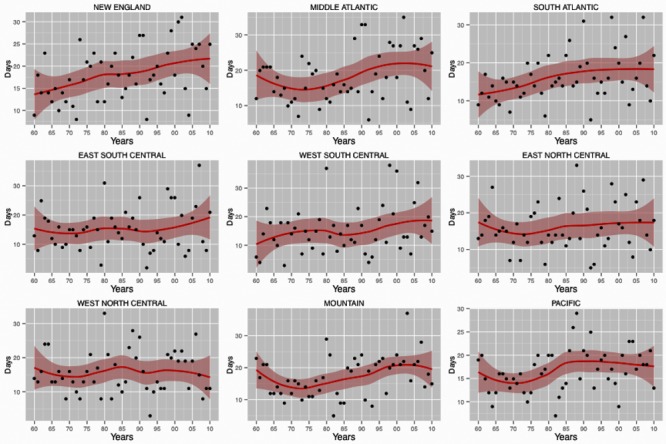
Temporal trend in extreme heat events across census division for the 1960–2010 periods.

The final extreme heat event dataset consisted of 3,109 counties over 51 years (1960 to 2010) located in the continental US (Table 1). In general, we observed significantly higher frequency of extreme heat events during the 1990s and 2000s compared to the reference period (1960–1989). This trend was consistent across season, 2006 urban-rural classifications and most Census divisions, with few exceptions. Within the two time periods (1990s and 2000s) the large central metro areas observed higher number extreme heat events compared to small metro and micropolitan areas. We also found an increasing trend in extreme heat events that varied considerably by area of the country, with the most pronounced trend observed for the New England, Middle Atlantic and Mountain divisions with lesser increases in the East and West North Central divisions (Fig 1). Interactions between time periods and Census divisions, ENSO, and Seasons were found to be highly significant and justified the stratification of the analysis by time period.

**Table 1:** County-level annual frequency of extreme heat events (mean (standard deviation, SD)), excluding Alaska and Hawaii.

		No. Counties	Time Period		
			1960–1989	1990–1999	2000–2010
*Contiguous US*		3109	15.2 (1.2)	16.5 (6.2)^+^	18. 2 (7.7)^+^
*Season*					
	Autumn	3109	3.7 (0.4)	4.1 (1.7)^+^	4.9 (2.0)^+^
	Winter	3109	3.9 (0.4)	5.1 (1.6)^+^	4.5 (1.6)^+^
	Spring	3109	3.8 (0.4)	3.6 (1.6)^‡^	4.4 (2.2)^+^
	Summer	3109	3.6 (0.4)	3.8 (2.7)^+^	4.4 (3.4)^+^
*County Urban-Rural Classification*					
	Large central metro	63	15.2 (1.4)	20.9 (6.2)^+^	19.7 (6.8)^+^
	Large fringe metro	354	15.1 (1.4)	17.8 (6.2)^+^	18.5 (8.0)^+^
	Medium metro	329	15.2 (1.2)	17.7 (7.4)^+^	19.2 (8.8)^+^
	Small metro	340	15.0 (1.2)	17.0 (6.2)^+^	18.0 (9.0)^+^
	Micropolitan	688	15.1 (1.2)	16.1 (6.3)^+^	18.0 (7.3)^+^
	Non-core	1335	15.2 (1.2)	15.8 (5.6)^+^	18.0 (7.3)^+^
*Census Division*					
	New England	67	16.3 (0.5)	19.9 (5.8)^+^	21.6 (8.4)^+^
	Middle Atlantic	150	16.1 (0.5)	21.4 (5.2)^+^	21.7 (6.0)^+^
	South Atlantic	589	14.5 (1.4)	18.3 (7.8)^+^	17.9 (10.6)^+^
	East South Central	364	14.7 (1.0)	14.6 (5.0)	17.2 (6.2)^+^
	West South Central	470	14.1 (1.2)	15.7 (6.7)^+^	18.4 (7.8)^+^
	East North Central	437	15.6 (1.0)	16.3 (3.6)^+^	17.4 (4.4)^+^
	West North Central	618	15.9 (0.8)	14.2 (3.3) ^‡^	16.7 (4.2) ^+^
	Mountain	281	15.4 (0.8)	18.0 (8.1)^+^	21.5 (11.0)^+^
	Pacific	133	15.8 (0.7)	18.2 (5.7)^+^	18.2 (6.1)^+^

^+^Significantly higher than the baseline (1960–1989) period(Pvalue <0.05).

^‡^ Significantly lower than the baseline (1960–1989) period(Pvalue <0.05)

Table 2 provides the frequency of extreme heat events stratified by phases of ENSO for the 3 time periods (1960–1989, 1990–1999, and 2000–2010), across season, 2006 urban rural classification, and Census Division. In general, La Niña periods were characterized by significantly higher frequency of extreme heat events while El Niño periods showed significantly lower frequency of extreme heat events for all seasons, 2006 urban-rural classifications, and most Census divisions when compared to the ENSO Neutral years. A noted exception to this pattern appeared in the 1990–1999 when the frequency of extreme heat events during El Niño were not lower than those observed during the ENSO neutral period, with some census divisions such as New England, Middle Atlantic, East/West North Central, Mountain and Pacific regions observing higher frequency of extreme heat events. Exceptions were also noted for winter of 1990s and 2000s, as well as summer of 1990s.

**Table 2:** County-level annual frequency of extreme heat events (mean (SD)) overall and by season, urbanization and Census Division, across decades and ENSO periods.

		No. Counties	Time Periods								
			1960–1989 (Baseline)			1990–1999			2000–2010		
			Neutral	La Niña	El Niño	Neutral	La Niña	El Niño	Neutral	La Niña	El Niño
*Contiguous US*		3109	15.1 (2.3)	17.8 (3.0)^+^	12.6 (2.1)^‡^	14.7 (6.2)	22.8 (11.0)^+^	15.3(7.3)^+^	17.8 (8.9)	22.3 (9.1)^+^	13.0 (7.6)‡
*Season*											
	Autumn	3109	16.0 (3.8)	16.0 (4.3)	13.0 (3.1)^‡^	13.9 (7.2)	21.6 (11.4)^+^	14.1 (8.6)	19.4 (12.2)	24.6 (11.0)^+^	11.8 (6.9)^‡^
	Winter	3109	15.5 (2.6)	16.2 (3.8)^+^	14.1 (4.9)^‡^	15.6 (7.1)	9.0 (11.1)^‡^	13.7 (9.0)^‡^	18.2 (9.1)	15.1 (12.0)^‡^	19.0 (13.0)
	Spring	3109	13.6 (3.2)	19.0 (6.8)^+^	12.1 (4.3)^‡^	12.5 (10.0)	26.7 (24.3)^+^	9.8 (11.4)^‡^	15.6 (14.5)	25.4 (23.1)^+^	14.8 (13.8)
	Summer	3109	15.6 (3.7)	20.1 (6.0)^+^	11.5 (3.4)^‡^	16.4 (8.3)	27.2 (11.5)^+^	22.2 (8.9)^+^	18.7 (8.6)	21.1 (8.9)^+^	9.4 (8.1)^‡^
*County Urban-Rural Classification*											
	Large central metro	63	15.0 (2.1)	18.0 (2.9)^+^	12.7 (2.1)^‡^	19.8 (6.7)	25.0 (10.0)^+^	20.0 (8.1)	19.6 (7.8)	22.8 (7.7)^+^	15.2 (7.4)^‡^
	Large fringe metro	354	14.6 (2.3)	18.7 (3.0)^+^	12.3 (2.0)^‡^	15.9 (5.8)	23.1 (11.2)^+^	17.6 (8.0)^+^	17.6 (9.0)	23.3 (9.2)^+^	13.1 (7.2)^‡^
	Medium metro	329	15.0 (2.1)	18.2 (3.1)^+^	12.6 (2.1)^‡^	16.2 (7.8)	24.1 (11.8)^+^	15.7 (7.9)	18.2 (9.6)	23.8 (10.1)^+^	14.2 (8.7)^‡^
	Small metro	340	14.8 (2.3)	17.9 (3.1)^+^	12.6 (2.2)^‡^	15.0 (5.8)	23.4 (12.3)^+^	15.9 (7.4)	17.6 (10.2)	22.0 (10.2)^+^	12.7 (8.7)^‡^
	Micropolitan	688	15.1 (2.3)	17.7 (3.1)^+^	12.6 (2.2)^‡^	14.2 (6.3)	22.5 (11.0)^+^	14.7 (7.2)	17.5 (8.4)	22.2 (8.8)^+^	12.8 (7.4)^‡^
	Non-core	1335	15.4 (2.3)	17.5 (2.8)^+^	12.6 (2.1)^‡^	13.9 (5.7)	22.4 (10.3)^+^	14.5 (6.6)	17.9 (8.6)	21.7 (8.6)^+^	12.7 (7.3)^‡^
*Census Division*											
	New England	67	15.4 (0.9)	18.9 (1.9)^+^	15.4 (1.4)	17.9 (5.2)	22.7 (8.4)^+^	21.5 (6.9)^+^	22.1 (9.5)	24.1 (8.7)^+^	16.9 (6.5)^‡^
	Middle Atlantic	150	15.0 (1.1)	20.5 (1.6)^+^	13.6 (1.1)^‡^	18.9 (4.8)	23.4 (8.3)^+^	24.4 (6.2)^+^	21.9 (6.8)	25.6 (6.6)^+^	15.5 (5.0)^‡^
	South Atlantic	589	13.8 (2.0)	19.3 (2.4)^+^	11.2 (1.9)^‡^	17.4 (7.7)	24.2 (12.8)^+^	15.4 (9.2)^‡^	16.3 (11.1)	22.6 (12.0)^+^	14.2 (9.6)^‡^
	East South Central	364	14.5 (1.9)	17.9 (3.0)^+^	11.9 (2.0)^‡^	12.0 (5.0)	25.4 (9.7)^+^	11.3 (5.3)	13.3 (5.9)	26.5 (8.9)^+^	11.0 (5.3)^‡^
	West South Central	470	13.7 (2.2)	14.9 (2.2)^+^	14.1 (2.2)^+^	13.0 (6.4)	30.3 (14.0)^+^	9.9 (4.9)^‡^	15.6 (7.8)	25.1 (9.0)^+^	13.7 (8.3)^‡^
	East North Central	437	14.6 (1.8)	20.1 (1.9)^+^	12.9 (1.7)^‡^	14.2 (3.7)	19.9 (6.1)^+^	17.5 (4.1)^+^	17.6 (6.0)	21.6 (4.9)^+^	10.8 (4.4)^‡^
	West North Central	618	16.7 (1.5)	18.0 (1.9)^+^	12.3 (1.5)^‡^	12.4 (3.2)	17.5 (5.6)^+^	15.2 (4.4)^+^	17.8 (5.7)	19.7 (4.8)^+^	10.2 (4.0)^‡^
	Mountain	281	17.6 (1.9)	14.9 (2.5)^‡^	11.8 (1.8)^‡^	16.1 (7.8)	23.1 (12.0)^+^	17.8 (8.1)^+^	24.7 (12.3)	20.0 (11.2)^‡^	17.3 (11.4)^‡^
* *	Pacific	133	17.2 (1.4)	15.0 (2.3)^‡^	14.0 (2.3)^‡^	18.1 (6.6)	16.6 (7.0)^‡^	19.6 (5.7)^+^	22.9 (7.4)	13.7 (7.1)^‡^	15.9 (6.6)^‡^

^+^Significantly higher than the ENSO Neutral period (P_value_ <0.05).

^‡^ Significantly lower than the ENSO Neutral period(P_value_ <0.05)

Table 3 presents the results from three negative binomial GEE models of monthly frequency of extreme heat events stratified by three time periods: 1960–1989, 1990–1999, and 2000–2010. Compared to ENSO neutral periods, El Niño periods were associated with significantly fewer events at the national scale, ranging from 9% fewer (estimated e^β^ = 0.91, p<0.001) during the 1990s to 24% fewer (estimated e^β^ = 0.76, p<0.001) during the 2000s, after adjusting for season, 2006 urban-rural classification and Census division (Table 3). By comparison, La Niña periods were associated with as much as 29% higher frequency of extreme heat events at the national level (estimated e^β^ = 1.29, p<0.001 for 1990–1999 & 2000–2010). For the 1990s and 2000s, counties that were large metropolitan areas based on the 2006 urban-rural classification tended to have a higher frequency of extreme heat events (estimated e^β^ >1.0) compared to non-core counties; although, this urban-rural difference was statistically significant only during the 1990s (p<0.05). Compared to New England, the other Census divisions of the country had significantly fewer differences in the extreme heat events for each of the three time periods (estimated e^β^ for all Census divisions <1.0), with the exception of the Middle Atlantic division.

**Table 3:** Relative percent change in extreme heat events, by time period, for the continental United States, excluding Alaska and Hawaii.

Factors		1960–1989	1990–1999	2000–2010
ENSO				
	Neutral	Reference		
	El Niño	0.85^‡^	0.91^‡^	0.76^‡^
	La Niña	1.17^‡^	1.29^‡^	1.29^‡^
Season				
	Autumn	Reference		
	Winter	1.04^‡^	1.28^‡^	0.88
	Spring	1.03^‡^	1.01	1
	Summer	0.98^‡^	0.91^‡^	1
Urbanization				
	Large central metro	0.99	1.22^‡^	1.05
	Large fringe metro	0.99	1.06^‡^	1.01
	Medium metro	1	1.06^‡^	1.04**
	Micropolitan	0.99	0.99	0.99
	Small metro	0.99	1.04**	1
	Non-core	Reference		
Census Division				
	New England	Reference		
	Middle Atlantic	0.98	1.07*	1
	South Atlantic	0.88^‡^	0.92^‡^	0.83^‡^
	East South Central	0.9^‡^	0.72^‡^	0.77^‡^
	West South Central	0.87^‡^	0.77^‡^	0.84^‡^
	East North Central	0.95^‡^	0.82^‡^	0.8^‡^
	West North Central	0.97**	0.72^‡^	0.78^‡^
	Mountain	0.94^‡^	0.91^‡^	0.99
	Pacific	0.97	0.92**	0.85^‡^

The coefficients are from the negative binomial GEE model described in the text.

*p < .05

** p < .005

‡ p < .001

The analysis for continental US were further broken down by Census division (Table 4). Overall, the Census division results agreed with the country level analysis presented in Table 3 with few noted exceptions. For example, compared to ENSO neutral periods, El Niño years were associated with significantly lower frequency of extreme heat events across Census divisions during 1960–1989 and 2000–2010 period. However, during 1990–1999, El Niño years were associated with increased frequency of extreme heat events compared to ENSO neutral years in several Census divisions (New England, Mid Atlantic and the Pacific divisions). La Niña periods were associated with a higher frequency of extreme heat events than ENSO neutral periods across most Census divisions; with the largest effect (75%) observed for the West South Central division during the 1990–1999 time periods. An exception to this pattern was in the Pacific division, where the La Niña period was associated with 15% lower frequency of extreme heat events compared to the ENSO neutral period during 2000–2010 (estimated e^β^ = 0.85, p<0.001).

**Table 4:** Relative percent change in count of extreme heat events, by Census Division (excluding Alaska and Hawaii).

Period	Factors		New England	Middle Atlantic	South Atlantic	East South Central	West South Central	East North Central	West North Central	Mountain	Pacific
**1960–1989 (Baseline)**											
** **	ENSO										
		Neutral^#^	1.00	1.00	1.00	1.00	1.00	1.00	1.00	1.00	1.00
		El Niño	0.94**	0.86‡	0.8‡	0.82‡	1.02**	0.82‡	0.81‡	0.81‡	0.9‡
		La Niña	1.13‡	1.24‡	1.31‡	1.23‡	1.04‡	1.27‡	1.15‡	1.04‡	0.99
	Season										
		Autumn^#^	1.00	1.00	1.00	1.00	1.00	1.00	1.00	1.00	1.00
		Winter	1.02	1.06**	1.05‡	1.04**	1.05‡	1.06‡	1.03**	1.01	0.96*
		Spring	1.05	1.08‡	1.06‡	1.03*	1.04*	1.05‡	1.01	1.02	1.01
		Summer	0.96	0.95*	1.03**	1.00	0.98	0.95‡	0.97**	0.92‡	0.97
	Urbanization										
		Large central metro	1.00	1.02	1.00	0.95	0.95	0.97	0.95	0.98	1.00
		Large fringe metro	1.01	1.00	1.02	0.98	0.94*	0.97	1.01	0.98	0.98
		Medium metro	0.99	1.01	1.01	0.98	1.01	1.01	1.00	0.99	1.00
		Micropolitan	1.00	1.00	1.00	0.99	1.00	0.99	0.99	0.98	0.99
		Small metro	0.99	0.99	1.01	0.98	0.97	0.97	1.01	0.98	0.98
** **	** **	Non-core^#^	1.00	1.00	1.00	1.00	1.00	1.00	1.00	1.00	1.00
**1990–1999**											
** **	ENSO										
		Neutral^#^	1.00	1.00	1.00	1.00	1.00	1.00	1.00	1.00	1.00
		El Niño	1.1**	1.15‡	0.89‡	0.81‡	0.71‡	1.01	1.01	1.00	1.08‡
		La Niña	1.07*	1.04	1.23‡	1.57‡	1.75‡	1.19‡	1.12‡	1.17‡	0.91
	Season										
		Autumn^#^	1.00	1.00	1.00	1.00	1.00	1.00	1.00	1.00	1.00
		Winter	1.63‡	1.68‡	1.29‡	1.13‡	1.3‡	1.64‡	1.32‡	0.87‡	0.86‡
		Spring	1.16**	1.2‡	1.04*	0.94*	1.32‡	1.11‡	0.8‡	0.86‡	0.94
		Summer	1.2‡	1.29‡	1.21‡	0.87‡	1.17‡	0.89‡	0.42‡	0.76‡	0.84‡
	Urbanization										
		Large central metro	1.05	1.14*	1.39‡	1.31*	1.34**	1.13	1.01	1.2*	1.15*
		Large fringe metro	1.11	1.03	1.15‡	1.08	1.09	0.99	0.93	0.99	1.08
		Medium metro	1.08	1.02	1.11**	1.1*	0.9*	1.06*	0.97	1.33‡	1.00
		Micropolitan	0.89*	1.06	0.9**	1.03	0.97	1.00	1.00	1.05	1.09*
		Small metro	0.91	1.07	1.02	1.07	1.08*	1.03	0.99	1.05	1.09
** **	** **	Non-core^#^	1.00	1.00	1.00	1.00	1.00	1.00	1.00	1.00	1.00
**2000–2010**											
** **	ENSO										
		Neutral^#^	1.00	1.00	1.00	1.00	1.00	1.00	1.00	1.00	1.00
		El Niño	0.82‡	0.74‡	0.79‡	0.7‡	0.87‡	0.69‡	0.69‡	0.9‡	0.97
		La Niña	1.2‡	1.27‡	1.27‡	1.65‡	1.37‡	1.3‡	1.21‡	1.02	0.85‡
	Season										
		Autumn^#^	1.00	1.00	1.00	1.00	1.00	1.00	1.00	1.00	1.00
		Winter	1.14‡	1.21‡	0.96*	0.8‡	1.07‡	0.85‡	0.82‡	0.64‡	0.71‡
		Spring	1.25‡	1.09**	1.01	0.96*	1.39‡	0.82‡	0.72‡	1.15‡	1.3‡
		Summer	1.27‡	1.35‡	1.25‡	1.16‡	1.18‡	0.7‡	0.54‡	1.22‡	1.05
	Urbanization										
		Large central metro	0.98	1.13*	1.01	1.01	1.28*	0.91	1.00	0.85*	1.19**
		Large fringe metro	1.10	1.08	1.02	1.04	1.04	0.91**	0.89**	1.27‡	0.98
		Medium metro	1.02	1.1*	1.01	0.98	1.03	0.97	0.99	1.45‡	1.11
		Micropolitan	0.94	1.08	0.91**	1.03	0.97	0.99	0.98	1.07*	1.09
		Small metro	0.93	1.14*	0.98	1.01	1.06	0.93**	0.96	0.93*	1.12*
** **	** **	Non-core^#^	1.00	1.00	1.00	1.00	1.00	1.00	1.00	1.00	1.00

*p < .05

** p < .005

‡ p < .001

^#^Reference Category

**Fig 2:**
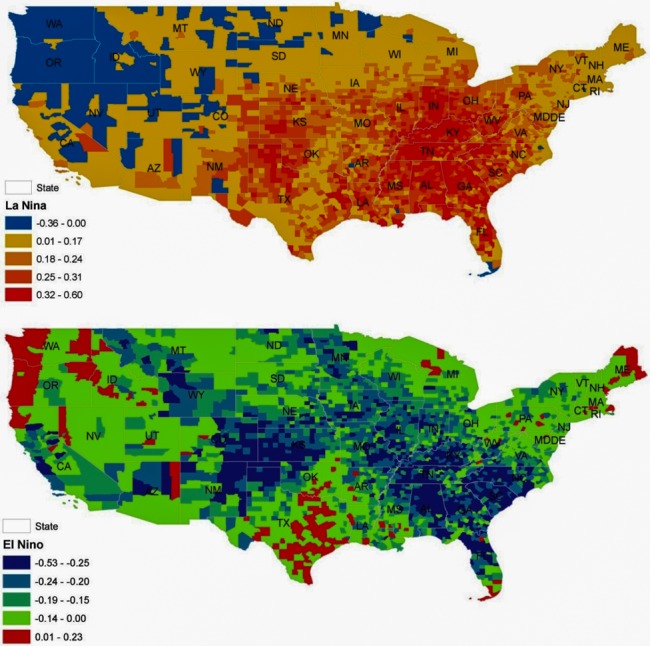
Relative percent change in monthly total extreme heat events for La Niña and El Niño months in 1960–2010 compared to ENSO Neutral months, adjusted for seasonal and 2006 land-use classification type.

Spatial heterogeneity in these findings was further investigated using the county level regression coefficients (Fig 2). The findings presented in Fig 2 are in agreement with the results of the divisional model (Table 4), but the finer county level resolution allows for the identification of additional counties whose results were masked in the divisional level analysis (e.g., selected counties in TX and ME experienced larger percent changes in frequency of extreme heat events during El Niño periods). We conducted sensitivity analysis using 1990 NCHS Urban-Rural Classification Scheme for Counties instead of the 2006 schemes; however this did not change our conclusions (results not shown).

## Discussion

We developed a generic surrogate exposure metric (extreme heat events) based on climatology that has a broad spatial coverage for the contiguous US with a county level geographic resolution. This exposure metric can correspond to county-level end-points, including many health outcome data such as behavioral risk factor surveillance system (BRFSS), Centers for Medicare and Medicaid Services (CMS), healthcare cost and utilization project (HCUP), CDC’s Public Health Tracking data and others. We assessed the exposure metric for its ability to capture the ENSO events, while controlling for other temporal, seasonal, divisional, and urban-rural classification influences. The results showed the ability of the exposure metric to capture salient features of climate variability and change (long term change in the frequency of extreme heat events) including the effect of natural variability such as El Niño-Southern Oscillation (ENSO) patterns that have distinct heterogeneous effects across geographical regions. We also demonstrated how the exposure metric could provide flexibility in spatial and temporal aggregation of exposure—an ideal attribute for epidemiological studies. Our county level approach enables a straightforward linkage of the exposure metric to many publically available national health outcome data collected at the county level, facilitating investigations of the possible impacts of climate change on chronic health outcomes [24–26].

The threshold method we used has been used for defining extremes in studies looking at temperature and precipitation extremes; however, exceedences have not been quantified on a county level for the entire US [27–30]. The metric we developed captured the local impacts of ENSO. This oscillation between warm (El Niño) and cold (La Niña) conditions in the equatorial Pacific Ocean can alter weather patterns and latent heat release into the atmosphere. Such changes lead to widespread remodeling in atmospheric circulation patterns far removed from the Pacific Ocean [31]. ENSO events have been linked to droughts, rainfall and the alteration of temperature and sunlight availability across the globe [32–34]. In North America, the statistically significant relationships between ENSO and seasonal temperature extremes have occurred mostly in winter [35]. In some divisions and times of year, El Niño and La Niña conditions modify the probabilities of very warm or very cold seasons [35]. The effects of climate change can manifest through natural forcing systems such as ENSO [36]. Globally, ENSO impacts are largely symmetric. The warm state (El Niño) is generally associated with increased precipitation and cooler temperature anomalies and the cold state (La Niña) changes the sign of the anomalies, to a decrease in precipitation and increase in temperature [19]. The known temperature-related impacts of ENSO were expressed in the results of our analysis. Though it should be noted that the surface expressions of the ENSO anomalies in the tropical Pacific are alleged to have changed since 2000 (albeit with similar onsets [37]) and the interconnectedness of these events (teleconnections) over the US appear to have changed [38]. On a continental scale, we found that La Niña periods were consistently associated with an increase in extreme heat events and El Ni**ñ**o periods led to a decrease in extreme heat events. In certain region of the country, the magnitude as well as direction of the associations between ENSO periods and extreme heat events differed from this trend. In this context, the effects of climate change may be local for health endpoints that may manifest via local weather changes [39,40].

Land use factors are an important contributor to divisional climate. Urbanization affects divisional climates through changes in surface energy and water balance. The change in land use can alter the effects of net radiation through the division of energy into sensible and latent heat, and the partitioning of precipitation into soil water, evapotranspiration and runoff [37]. The urban “heat island” effect is an extreme case of how land use modifies divisional climate [41,42]. Previous studies have suggested that a major portion of the reduction in diurnal temperature range observed during the last several decades to urbanization and other land use changes [43,44]. In congruence with available literature, using the 2006 urban-rural classification, we found that more urbanized areas generally experienced relatively high proportional change in extreme heat events compared to the less urbanized areas. However, this pattern was not consistent across Census divisions and was only present during the latter 2 decades. These results may be due, in part, to the classification scheme used in this analysis. This scheme was developed based on the 2006 census statistics and applied in our study for time periods that span more than 4 decades prior. However, sensitivity analyses using the 1990 census scheme produced similar results.

More attention has been paid to the effects of hot temperature anomalies in the summer and spring particularly because these changes can have an impact on biotic factors (e.g., pollen) and industrial air pollution along with heat waves [45–49]. Using the metric, we identified larger differences in extreme heat events occurring during the winter, spring and summer months on a continental scale. Yet, at the divisional level, the patterns differ considerably, with the New England and Middle Atlantic divisions experiencing the largest differences in extreme heat events during winter and lowest level during autumn. In the Mountain and Pacific divisions, the largest differences in extreme heat events were observed during spring and lowest level observed during winter season.

Overall, the exposure metric captured subtle variability across geographic division, season, and urban-rural categorization. More importantly, the exposure metric was sensitive to large scale phenomenon such as ENSO that are known to govern local weather patterns. As stated previously, the flexibility of this exposure metric lends itself to epidemiological studies of both infectious and chronic diseases. For example, in a recent study investigating the link between changing climate and Salmonellosis, Jiang et al. (2015) showed that the frequency of extreme heat and precipitation event was directly related to increased risk of Salmonellosis in Maryland, and that the risk was more pronounced among the coastal communities compared to inland communities [50]. Since the precise date of disease onset in the Jiang et al. (2015) was not known, the authors linked monthly count of Salmonellosis with number of extreme heat and precipitation event on the same month and employed negative binomial regression for the statistical analysis. In the instances where the precise date of onset is known (e.g., hospitalization for asthma, or stroke), investigators can use case-crossover approach looking at presence/absence of extreme events in the case period compared to control period with adequate lag structure that are determined based on current knowledge about the disease etiology. In addition, the frequency of extreme heat events can also be used to investigate the spatio-temporal pattern of vector borne diseases (e.g., Lyme disease) that are sensitive to temperature changes. Previous studies have shown that the frequency as well as intensity of extreme events will continue to rise in the near future [51,52]. The exposure metric we have presented in this manuscript allows investigators to document how increases in the frequency of extreme heat event impacts human health.

## Conclusion

We report on the development of a novel temperature-related exposure metric and quantify its ability to capture small and large changes in climatic variability across the US and over time. Findings from this study suggest that natural modes of forcing, seasonality, urban-rural classification, and division of country have an impact on the number extreme heat events recorded. We observed that the increases in frequency of extreme heat events differ across the geographical region and time periods. Likewise, we observed higher frequency of extreme heat events during La Niña period and lower frequencies during the El Niño. At regional level, exceptions to this trend were noted for El Niño years in selected geographical areas. This county level exposure metric generated based on location specific climatology data is versatile and can be easily extended to developing metrics for different time periods and county based geographic aggregations. To facilitate research in this area, we will make this exposure metric freely available to potential users through a web portal.
